# Promoting a Patient-Centered Understanding of Safety in Acute Mental Health Wards: A User-Centered Design Approach to Develop a Real-Time Digital Monitoring Tool

**DOI:** 10.2196/53726

**Published:** 2024-04-12

**Authors:** Gemma Louch, Kathryn Berzins, Lauren Walker, Gemma Wormald, Kirstin Blackwell, Michael Stephens, Mark Brown, John Baker

**Affiliations:** 1 School of Healthcare University of Leeds Leeds United Kingdom; 2 Yorkshire Quality and Safety Research Group Bradford Institute for Health Research Bradford United Kingdom; 3 Health Technology Assessment Unit, Applied Health Research Hub, Implementation and Capacity Building Team NIHR Applied Research Collaboration North West Coast University of Central Lancashire Preston United Kingdom; 4 School of Health and Psychological Sciences City, University of London London United Kingdom; 5 Thrive by Design Leeds and York Partnership NHS Foundation Trust Leeds United Kingdom; 6 Ayup Digital Leeds United Kingdom; 7 Social Spider CIC London United Kingdom

**Keywords:** patient safety, mental health, patient involvement, qualitative, digital innovation, real time, monitoring, safety, develop, development, design, perception, perceptions, prototype, evidence scan, interview, interviews, logic model, programme theory, dashboard, dashboards, interface

## Abstract

**Background:**

Acute mental health services report high levels of safety incidents that involve both patients and staff. The potential for patients to be involved in interventions to improve safety within a mental health setting is acknowledged, and there is a need for interventions that proactively seek the patient perspective of safety. Digital technologies may offer opportunities to address this need.

**Objective:**

This research sought to design and develop a digital real-time monitoring tool (WardSonar) to collect and collate daily information from patients in acute mental health wards about their perceptions of safety. We present the design and development process and underpinning logic model and programme theory.

**Methods:**

The first stage involved a synthesis of the findings from a systematic review and evidence scan, interviews with patients (n=8) and health professionals (n=17), and stakeholder engagement. Cycles of design activities and discussion followed with patients, staff, and stakeholder groups, to design and develop the prototype tool.

**Results:**

We drew on patient safety theory and the concepts of contagion and milieu. The data synthesis, design, and development process resulted in three prototype components of the digital monitoring tool (WardSonar): (1) a patient recording interface that asks patients to input their perceptions into a tablet computer, to assess how the ward feels and whether the direction is changing, that is, “getting worse” or “getting better”; (2) a staff dashboard and functionality to interrogate the data at different levels; and (3) a public-facing ward interface. The technology is available as open-source code.

**Conclusions:**

Recent patient safety policy and research priorities encourage innovative approaches to measuring and monitoring safety. We developed a digital real-time monitoring tool to collect information from patients in acute mental health wards about perceived safety, to support staff to respond and intervene to changes in the clinical environment more proactively.

## Introduction

### Overview

High levels of patient safety incidents are reported within mental health services. Between April 2020 and March 2021, a total of 300,703 incidents were reported in England, often involving self-harming behavior and disruptive, aggressive behavior [1]. Patient safety within mental health services is recognized as a priority within the National Health Service (NHS) Patient Safety Strategy 2021 update [2] and is a key research priority within the extant literature [3-6].

Supporting patients and families to be partners in care safety can be viewed as both a logical and moral imperative [7]. Furthermore, understanding the patient perspective is an important patient safety priority in the mental health field [4]. Recognizing the limitations of patient safety incident reporting systems, research evidence suggests that patients and families are a valuable source of safety intelligence that can inform service improvement in acute [8-10] and primary care settings [11-13]. Indeed, our previous research directly addressed this priority for mental health patient safety research, by evidencing that patients acknowledge the value of their potential involvement in interventions to improve safety and the need to develop interventions that proactively seek the patient perspective of safety [14,15].

In addition to understanding the patient perspective, there is also policy and research impetus to understand safety in real time, to shift from a reliance on retrospective patient safety measures [6,16], and a push toward embracing innovative approaches and digital solutions to safety challenges [6,17].

Considered together, these policy and research priorities provide the rationale for the development of a digital monitoring tool that captures the patient perspective of safety in real time, in an acute mental health ward setting.

### Theoretical and Conceptual Underpinning of the Digital Monitoring Tool

The theoretical foundation for the development of a digital real-time monitoring tool is embedded within the measurement and monitoring of safety (MMS) framework [16]. Previous research exploring the framework in practice acknowledged its potential to support a broader and richer approach to organizational safety [18,19]. Specifically, we focus on 1 domain of the framework—sensitivity to operations. This domain describes the need for a collective awareness by staff of the workings of the service and their ability to be sensitive and responsive to subtle changes and disturbances. This domain highlights the crucial but often overlooked activity of “monitoring” the safety of care as it is delivered in real time, and it is a domain where patients and families are recognized as potential key sources of information. Within the wider patient safety literature, the importance of safety “monitoring” as opposed to solely “measurement” and the potential of prospective clinical surveillance as a means of promoting safety within organizations have gained traction [20,21]. Supporting the idea of prospective clinical surveillance as a means of promoting safety is particularly important within the acute mental health care context, where fluctuations in the dynamic of the inpatient group and the interplay between patients, staff, and the environment can occur rapidly, with individual patient needs creating immediate knock-on effects for other patients, their quality of care, and their safety.

Two further concepts that informed our thinking and the development of the real-time digital monitoring tool are milieu and contagion. The concept of milieu is often aligned with the notion of ward atmosphere, which involves the interplay between the physical environment, social structures, and social interactions [22]. In this work, we conceptualize the milieu as being akin to the ward atmosphere. Safety may be related to tensions in the milieu of the ward [23], and improving the milieu may improve safety [24]. There is research to support the notion of a “contagion effect” of safety incidents (violence and self-harm behaviors) within an inpatient mental health ward setting [25], with evidence to suggest that patient aggression and self-harm behaviors do not occur at random intervals, but cluster temporally. Therefore, a key safety issue is the potential for 1 incident to increase the likelihood of further incidents occurring as a result of disturbed ward milieu and contagion.

Considering contagion and milieu alongside the domain sensitivity to operations of the MMS framework [16] underpinned our operationalization of how we might capture perceptions of safety in real time. A digital monitoring tool capturing how the ward is perceived by patients (ie, the milieu) at any given time may provide intelligence that supports staff to respond proactively to emerging safety issues and intervene earlier, which may prevent future incidents from occurring via disturbed ward milieu and contagion.

### Aim

The aim of this study is to design and develop a digital real-time monitoring tool that collects and collates information from patients in acute mental health wards about their perceptions of safety, in order to support staff in monitoring and improving the safety of the clinical environment.

### Approach

This paper focuses on the design and development of the digital monitoring tool. Two stages are presented: stage 1—a synthesis of exploratory research, existing research, and stakeholder engagement and stage 2—design and development. The methods and results are presented for each stage separately. The findings relating to the implementation and evaluation (qualitative) of the tool are reported elsewhere [26] and include further information about how the tool was operationalized in the implementation phase.

## Methods

### Stage 1: Exploratory Research, Existing Research, and Stakeholder Engagement Synthesis

#### Objective

This stage focused on bringing together and synthesizing key findings from our exploratory research, previous research, and early stakeholder engagement discussions. The purpose of this synthesis was to generate themes, preliminary ideas, and needs for the monitoring tool from multiple perspectives.

#### Activities and Analysis

We conducted (1) a systematic review [27] and evidence scan, (2) semistructured interviews with health professionals [28] and patients, and (3) stakeholder engagement sessions (see Textbox 1).

**Overview of activities contributing to data synthesis.**

**Systematic review and evidence scan**

- Systematic review of patient involvement in safety interventions in an acute mental health setting [27]:
  Included 52 articles
  Narrative synthesis
- Evidence scan focused on the application of digital technology in mental health contexts. Databases searched: CINAHL, PsycINFO, and Web of Science. Search terms—mental health AND digital technology AND safety, search date—November 5, 2020.
  Included 13 articles (see Multimedia Appendix 1)
  Narrative synthesis

**Semistructured interviews with health professionals and patients**

- Interviews with patients (n=8; 2 participants identified as female, and 6 as male) older than 18 years of age with current or recent experience (during the past 2 years) of being an inpatient in an acute mental health ward from 2 Mental Health Trusts in the North of England.
- Interviews with mental health professionals (n=17), methods and findings reported elsewhere [28].
  The interviews aimed to understand perspectives on safety issues and how patients and health professionals can contribute toward the measurement and monitoring of ward safety in an acute mental health setting.
  Reflexive thematic analysis [29,30]

**Stakeholder engagement sessions**

- Sessions held with 2 existing patient focused stakeholder groups via video conferencing software, hosted by a Mental Health Trust in the North of England in August 2020, focusing on:
  How to phrase or ask patients about whether wards feel safe or unsafe
  Language and how best to talk about patient safety (eg, words or phrases that should or should not be used)
  What to consider when developing digital interventions to be used in acute mental health wards

### Ethical Considerations

The interview study with health professionals received ethical approval from the University of Leeds, School of Healthcare Ethics Committee (HREC 19-028), and the interview study with patients received ethical approval from South Central—Berkshire B Research Ethics Committee (20/SC/0360). Prior to the interviews, researchers explained the purpose and process of the interview to the participant, read the consent form out loud, and recorded consent verbally. Interviews were audio recorded, anonymized, and transcribed professionally. Patients who were interviewed received a £10 (US $12.79) shopping voucher as a thank you for their time.

### Data Synthesis

We produced summary documents for the systematic review and evidence scan, ongoing interviews with patients and health professionals, and stakeholder engagement sessions. The findings were organized and reviewed through the lens of implications for the design and development phase and discussed on an ongoing basis between the core research team (including 2 co-investigators with lived experience), the project Steering Group, digital partners (Ayup Digital), and co-design partners (Thrive by Design).

### Stage 2: Design and Development

#### Objective

This stage was informed by stage 1 and focused on the design and development of a digital monitoring tool to enable a real-time understanding of the patient perspective of safety in acute mental health wards.

#### Activities and Analysis

This stage was significantly affected by the COVID-19 pandemic [31]; pragmatic adjustments were made to the original co-design plan to produce a feasible alternative in which a single NHS Trust permitted limited visits from a clinical member of the co-design team who was familiar with the local protocols.

At the outset of this stage, members of the core research team, digital partners, and co-design partners drew from the ongoing stage-1 synthesis to inform the basis of the subsequent activities. The reworked co-design activities included face-to-face discussions and opportunistic feedback from patients and staff during ward visits in 2 wards in the North of England. This stage also involved further stakeholder engagement sessions with the same 2 stakeholder groups described in stage 1. We used a collaborative, human-centered, and sprint-based or agile approach [32,33], incorporating techniques such as “personas” (typical users with specific characteristics including experience of disability and different levels of digital skills), storyboards (to elicit user goals), patient journey mapping (the path of an individual patient and health care staff member in a ward), user stories (to elicit specific user requirements), and prototyping (rapid creation of paper prototypes of a digital tool). The first cycle of activities generated requirements for the digital tool; subsequent activities tested and refined the tool as it developed and considered its use within the clinical workflow of the ward environment.

### Ward-Based Activities

#### Visit 1 (December 2020)

Draft ideas for the digital products were discussed, for example, wording, format, color, and how they would work in relation to existing systems. People wrote comments and provided feedback on all aspects of the design. From the staff perspective, we explored whether the visuals looked like anything already in use, to avoid unintended consequences arising from confusion.

- Ward A (adult acute ward caring for male patients aged 18-65 years): opportunistic feedback and discussion with 3 patients and 3 members of staff (clinical and administrative).
- Ward B (adult acute ward caring for female patients aged 18-65 years): opportunistic feedback and discussion with 5 patients and 12 members of staff (clinical and administrative); a total of 3 members of staff and 5 patients participated in a co-design workshop (approximately 40 minutes duration).

Once activities commenced in the wards, members of the core research team, digital partners, and co-design partners continued to meet fortnightly to interrogate and interpret the feedback on the digital product ideas in more detail, focusing on who would benefit, considering the “must haves,” and recording when there was consensus on what not to include. Discussions centered on how many of the “must haves” were realistic to implement, and alongside input from the project Steering Group fed into the development of the second iteration of the digital products.

#### Visit 2 (January 2021)

Discussions and activities focused on the refined prototype ideas for the digital products. For both Ward A and Ward B, multiple versions of the latest iteration of products were presented and feedback elicited, in addition to revisiting discussions from visit 1. People provided feedback on the wording, icons, colors, and representations, that is, was it what they imagined? From the staff perspective, the requirements of the staff dashboard were revisited, and from the patient and staff perspective, the acceptability of a public-facing ward interface was explored. As there was a high turnover of patients, many new patients were involved, although many of the same staff contributed, which enabled discussions from the first visits to be revisited.

- Ward A: opportunistic feedback and discussion with 5 patients and 3 members of staff (clinical and administrative).
- Ward B: opportunistic feedback and discussion with 6 patients and 5 members of staff (clinical and administrative).

Feedback from the ward-based discussions and activities was collated and prioritized using the Must Have; Should Have; Could Have; Won’t Have this time (MoSCoW) method [34], which then guided conceptual and technical development. Prototype ideas were refined following the prioritization exercise.

### Stakeholder Engagement

Sessions followed via video conferencing software with the same 2 stakeholder groups described previously in stage 1 (July 2021). The most recent prototype ideas were presented and discussed, focusing on (1) the design of the patient interface: Would you feel comfortable completing it? (2) Could you say why you would feel more or less comfortable? (3) Do you think completing a report on the ward atmosphere on a digital tablet would make you feel less or more distressed? and (4) public-facing ward interface: How would you feel about this being displayed in a ward?

## Results

### Stage 1: Exploratory Research, Existing Research, and Stakeholder Engagement Synthesis

The findings from the synthesis are organized around 7 themes with associated implications for the subsequent design and development phase (see Textbox 2). Supporting information for each theme is outlined in Tables S1-S7 in Multimedia Appendix 2.

**Themes and design and development implications.**

**Conceptualization of safety**

- Allow for a multifaceted conceptualization of safety.
- Consider how the word “safety” may be misinterpreted—provide a clear description.

**Anonymity**

- Anonymity essential.
- Reinforce feedback is anonymous and that everyone’s experience is important—all feedback valued.
- Give clear information about the use and purpose of collecting information via digital technology.

**Milieu (ward atmosphere), contagion, and incidents**

- The technology needs to be sensitive to subtle changes in a negative direction in order to anticipate the potential for an incident occurring.
- Explore whether the technology can be sensitive to context, for example, highlight when a safety incident has recently occurred.
- Location: potential for the technology to be sensitive to location within the ward, for example, increased anxiety or input activity by patients into the technology (prior to and after an incident).
- Night and day: the technology needs to recognize night and day as different contexts.
- The technology may need to account for or recognize ward profiles (eg, all male, all female, and mixed wards).
- The technology may need to account for or recognize differences between individual patients.

**Digital technology in the ward**

- Ensure the purpose of the monitoring tool is clearly described to patients and staff.
- The technology needs to be inclusive across a wide range of patients and digital abilities.
- Staff may need support with using the technology and with data interpretation.
- Consider multiple mechanisms for data collection other than a single device or reporting “point.”
- Consider other relevant systems in place for staff.

**Involving patients in understanding safety**

- Technology needs to incorporate the input of quantitative and qualitative data to give patients choice about the level of detail they provide.
- Explore using language or imagery that is universal and include a free text option to expand.
- Consider different levels of providing feedback, for example, level 1 reporting—a color or smiley face and level 2—more detailed information.
- Explore whether thresholds need to be built into the system to trigger alerts or action.
- Consider how the phrases used to describe safety converge with those used by other relevant organizations.
- Consider the location of data collection and timings.
- Consider the implications of the type of ward.

**Feeding data back**

- The technology and mechanism of feeding data back needs to work within existing trust and ward infrastructure.
- Explore frequency or timing of monitoring the data to ensure staff can be responsive.
- Consider approaches to displaying the data.
- Consider who can see the data and when.
- Staff may need support with data interpretation and action.

**Unintended consequences**

- Important to provide choice and freedom on when to provide feedback.
- May need to offer different levels of providing feedback.
- Consider how the technology sits alongside verbal information and feedback.

### Stage 2: Design and Development

#### Intervention Design and Description

##### Overview

The latest version of the digital monitoring tool, WardSonar (prototype 3), includes 3 components—a patient recording interface, a health professional dashboard, and a public-facing ward interface. Further information about the prioritization process and conceptual and technical development is provided in Multimedia Appendix 3.

##### Patient Recording Interface

**Figure 1:**
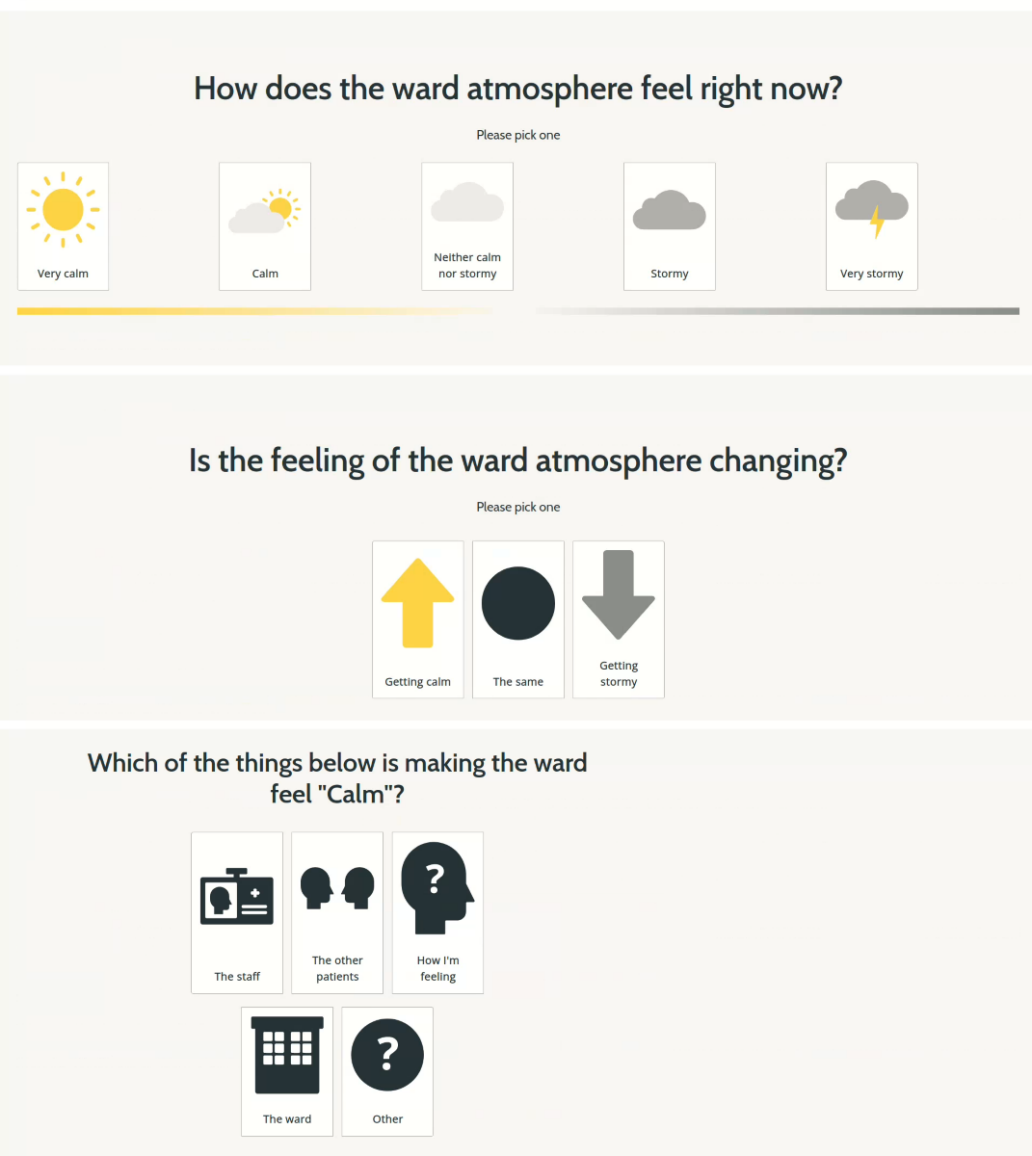
Prototype-3 patient recording interface.

The recording interface is an app accessed on a tablet device (see Figure 1). The interface provides brief background information and prompts patients to answer a series of questions. No patient data are collected, and all data collected on the interface are anonymized at the point of entry. The interface uses a weather analogy with questions, such as “How does the ward atmosphere feel right now?” (very calm to very stormy), as the co-design phase identified the need to provide different ways for people to be able to express how the ward was feeling, such as pictures and text. Therefore, free-text options were also included. The design and development phase highlighted that more options for how the ward was feeling would be beneficial to reflect that this is often more than “good, bad and okay” and that the feeling is more of a spectrum. Therefore, the interface includes 5 options to describe how the ward is feeling, and an additional question to indicate the direction things are moving in (ie, getting better, getting worse, or staying the same). Attention was paid to understanding the contextual constraints and pressures that patients may be experiencing (eg, location when entering data and current mental state at the time of data entry), and feedback from the design and development phase suggested that a reporting “point” could create negativity toward the area it is placed and prevent people from using it.

The idea of patients using their own mobile phones to provide feedback was also considered in the design and development phase. This was not progressed at this initial stage as not all patients have a smartphone or access to data, and from a technological development perspective, in the subsequent implementation phase, each ward required a secure login to access the app. Organizing this for each patient on either their own or a study-bought device would have required technological support in the wards, which would not have been feasible during the COVID-19 pandemic, and we were aiming for the approach to be implemented with minimal support. Therefore, in the subsequent implementation phase, it was decided that staff would supply patients with the tablet device (provided by the research study) optimally 3 times per day so patients could enter real-time safety perspectives. Staff supplying the tablet device to patients also addressed staff concerns about the security of the tablet devices and who would have responsibility for them.

##### Staff Dashboard

**Figure 2:**
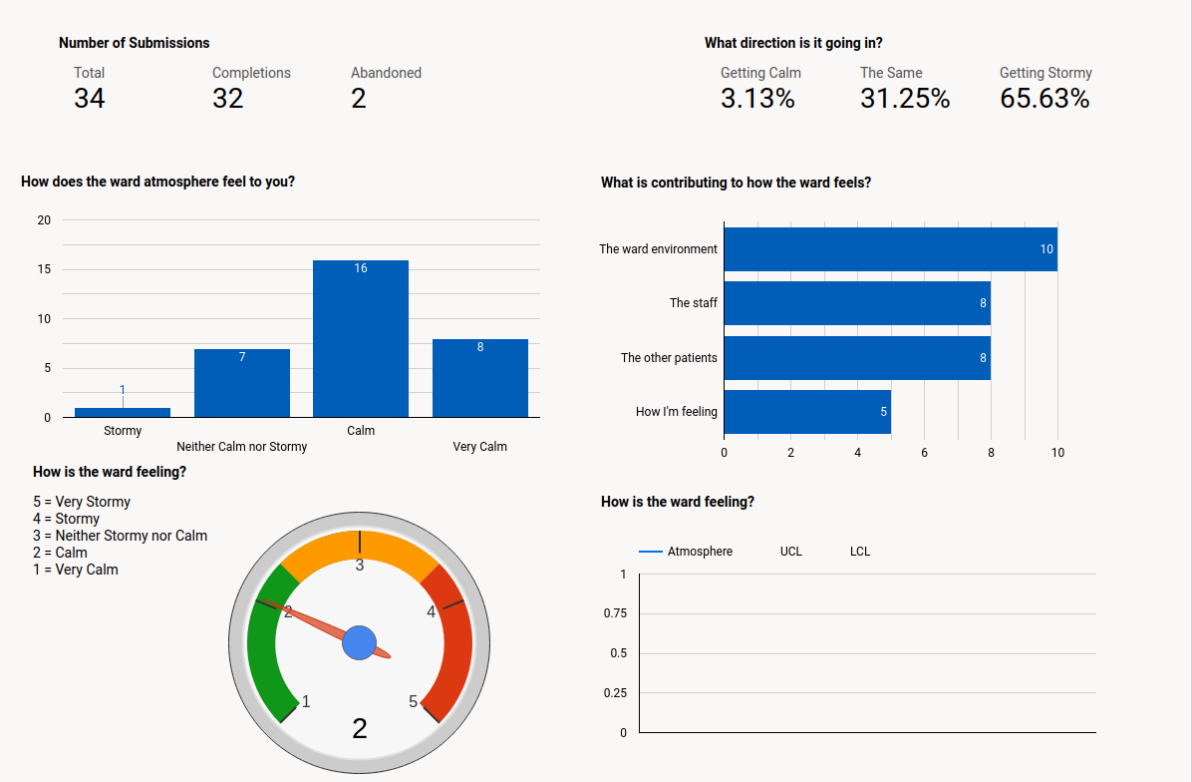
Prototype-3 staff dashboard.

The staff dashboard is the main interface used by staff to view data submitted by patients on a tablet device or desktop computer (see Figure 2). It is accessed in specific, authorized locations, such as the ward office, and provides real-time snapshot data and greater informational insights through the use of data visualizations. The co-design phase with staff, statisticians, and researchers resulted in the dashboard including barometer-style visualization of “How is the ward feeling,” statistical process control charts, graphs, and statistical metrics, and the functionality for monthly, weekly, daily, or shift aggregate data to be comparable to historic time periods.

##### Public-Facing Ward Interface

**Figure 3:**
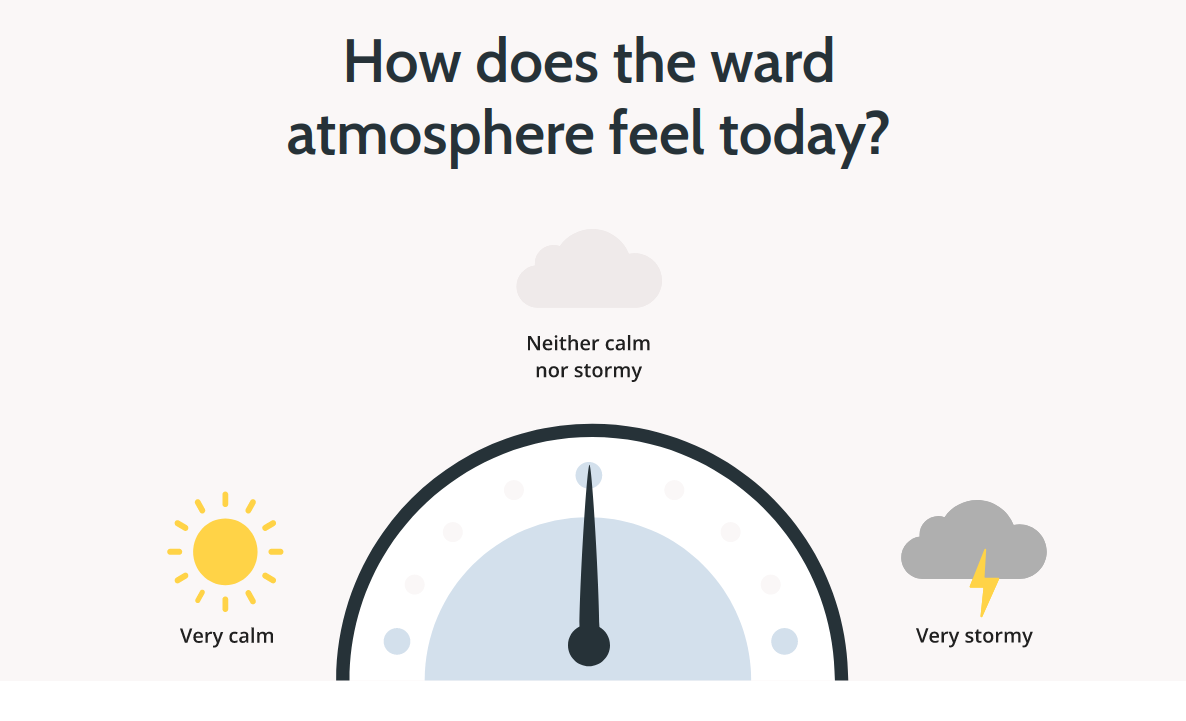
Prototype-3 public-facing ward interface.

The public-facing ward interface displays the average ward atmosphere rating for the current shift on, for example, a television screen, desktop computer monitor, or tablet device (see Figure 3). The outcome measure is displayed in the form of a barometer.

#### Stakeholder Engagement

The 2 groups were positive about the prototype-3 patient recording interface, in particular, the simplicity of the interface and the anonymity it provides. Overall, people felt the patient interface would be a safe space to report concerns. However, it was emphasized that this system should not replace reporting in person to staff. Regarding the question of factors affecting how the ward feels, there was consensus that it was important to be able to select more than 1 factor and this change was subsequently implemented. A number of potential unintended consequences were emphasized such as people being concerned that others were making reports about them; that the technology might trigger people’s symptoms; and that the ward interface could be confusing, as patients may think it referred to the weather outside. Of particular note were discussions around the potential for a poor ward atmosphere when a ward is understaffed, meaning there may be limited support from staff to circulate the device at these times. There were concerns raised around the accessibility and inclusivity of the patient recording interface which would need to be addressed in future iterations, for example, people who may experience barriers related to language or literacy, people with learning disabilities, and people with visual impairments. As potential unintended consequences were highlighted regarding the public-facing ward interface, this component of the tool did not progress to the subsequent implementation phase [26].

#### Technical Specifications

The digital products were built using open web standard technologies (eg, HTML5, CSS3, JavaScript, and PHP) in a componentized and scalable way, to allow modifications and future developments, and were based on the Government Digital Service Agile Delivery methodology framework and NHS standards. All digital products are hosted in a custom cloud environment built on top of the Amazon Web Services infrastructure. Data are recorded in real time and sent to an application programming interface. The interface was built to Web Content Accessibility Guidelines (WCAG) 2.1 AA [35] standards and is device agnostic.

Patients are able to input data via a device supplied by ward staff. Patient data are monitored through the staff dashboard by authorized members of staff with access via a tablet device or desktop computer. Any data input by patients are recorded via the app and is automatically sent back in real time to the central database. If Wi-Fi is temporarily unavailable in the ward, then the data that are input are stored locally on the device in offline mode. When the device comes back into the Wi-Fi range the data are automatically submitted to the central database. Further information about the technologies used and infrastructure is provided in Multimedia Appendix 4.

#### Logic Model and Program Theory

**Figure 4:**
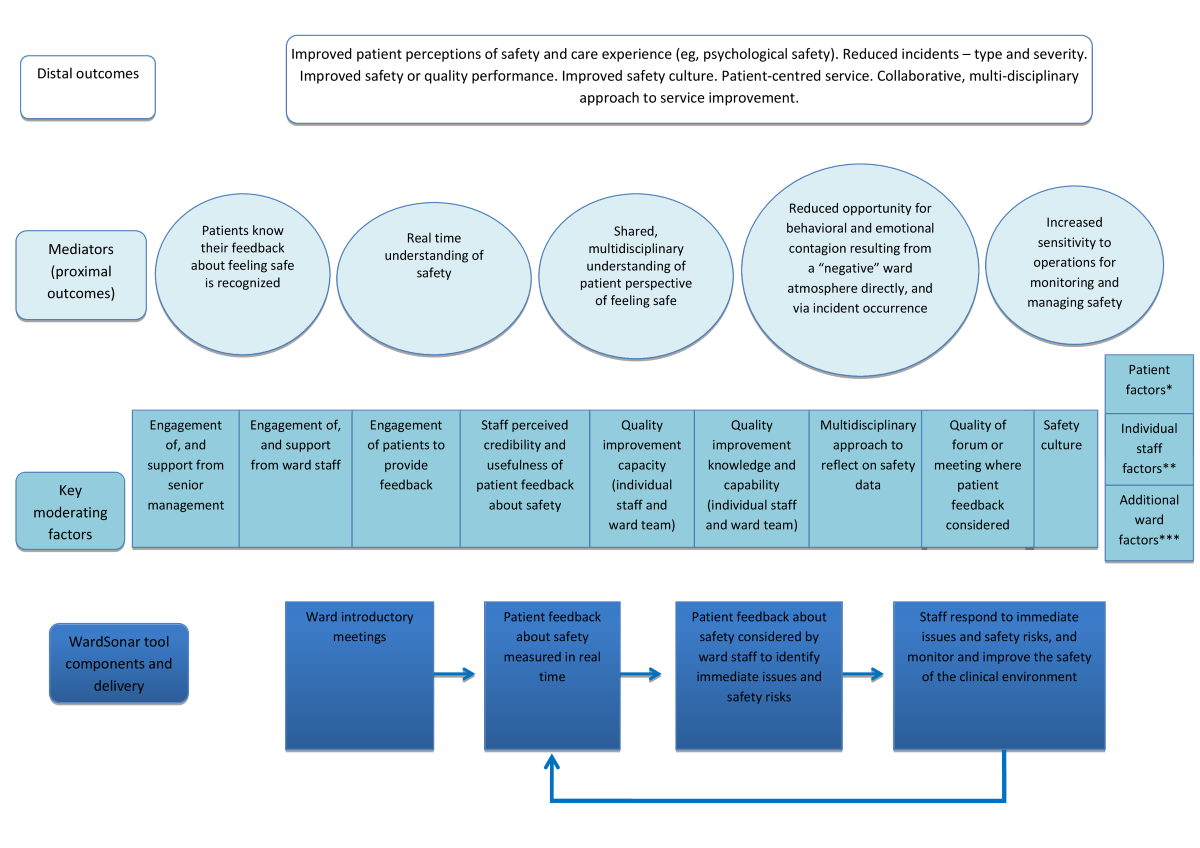
The logic model for the WardSonar monitoring tool. *For example, gender, ethnicity, disorder, and digital literacy; **for example, qualification and gender; ***for example, skill mix and staffing levels.

A logic model was generated to articulate how the various components of WardSonar related to each other (see Figure 4). In conjunction with the logic model, Multimedia Appendix 5 provides a narrative description of the program theory explaining how WardSonar might facilitate change in proximal and distal outcomes.

## Discussion

### Principal Findings

We successfully developed WardSonar, a theory-informed, patient-centered, real-time digital safety monitoring tool for acute mental health wards. The WardSonar monitoring tool aims to collect daily information from patients in acute mental health wards about perceived safety to support health professionals in monitoring and improving the safety of the clinical environment.

The process of design and development included synthesizing findings from a systematic review, evidence scan, interviews with patients and health professionals, and stakeholder engagement, to inform a cycle of design activities. The synthesis highlighted broad themes and potential unintended consequences to be explored further within the subsequent design phase. For instance, the importance of the tool allowing for a multifaceted conceptualization of safety, ensuring patient anonymity, being sensitive to subtle changes, and being inclusive across a wide range of patients and digital abilities.

The design and development process resulted in 3 components of the monitoring tool. First, a patient recording interface to assess how the ward feels. Patients were keen to have different ways to be able to express how the ward was feeling, including pictures, text, and being able to write in free text. The need to capture whether the direction is changing, that is, “getting worse” or “getting better” was felt to be really important from a health professional perspective. The second component was a staff dashboard with functionality to interrogate the data at different levels, and the third component was a public-facing ward interface. Potential unintended consequences and challenges were highlighted, for example, the public-facing ward interface being potentially triggering if displaying a negative ward atmosphere. Embedding the principles of WardSonar within the MMS framework [16], and within the domain sensitivity to operations specifically, has produced a safety monitoring tool that recognizes patients as a key source of safety information and facilitates health professionals being aware of subtle changes and disturbances.

### Implications for Research and Practice

The next step is for the WardSonar monitoring tool and its components to be implemented in practice, and for this to be accompanied by a robust evaluation. This will generate evidence around key questions pertaining to feasibility and acceptability, for example, is it feasible and acceptable to collect safety data from patients in mental health wards? and, how do staff use the data collected from patients? Such an evaluation may support further refinement of WardSonar, and over the longer term, would explore the assumptions articulated in the WardSonar logic model, for instance, our hypothesized distal outcomes, leading to a refined logic model and program theory in line with Medical Research Council guidance on developing and evaluating complex interventions [36].

A key feature of the design and development phase was the need for WardSonar to be accessible for people with different language and literacy needs and for it to be accessible for a range of digital abilities. Our most recent stakeholder engagement identified concerns around accessibility and inclusivity of the patient interface. Therefore, it will be essential for these concerns to be examined further in subsequent evaluation work to ensure future iterations of the WardSonar tool can be accessed equitably.

### Strengths and Limitations

To our knowledge, the WardSonar monitoring tool is the first system that actively seeks the perspective of safety from patients in real time for use within acute mental health wards. A key strength of this work is our theory-based approach and the multiple data sources brought into the design and development phase. A limitation of our work is the level of co-design and stakeholder engagement activity we were able to undertake, as our original plans had to be amended considerably due to the impact of the COVID-19 pandemic on the project.

### Conclusions

Recent patient safety policy and research priorities encourage innovative approaches to measuring and monitoring safety, and there is a need for a patient-centered understanding of safety in an acute mental health setting. At present, no interventions or tools exist to address this need. We developed a digital real-time monitoring tool to collect information from patients in acute mental health wards about perceived safety, to support health professionals to respond, and intervene to changes in the clinical environment more proactively. Further research is required to evaluate the implementation of WardSonar to further refine and improve this innovative approach.

## Appendix

Multimedia Appendix 1 Evidence scan included articles.

Multimedia Appendix 2 Supporting information for each theme.

Multimedia Appendix 3 Feedback following the ward visits and prioritization.

Multimedia Appendix 4 Technologies used and infrastructure.

Multimedia Appendix 5 WardSonar programme theory narrative description.

## Abbreviations

MMS: measurement and monitoring of safety
MoSCoW: Must Have; Should Have; Could Have; Won’t Have this time
NHS: National Health Service
WCAG: Web Content Accessibility Guidelines

## Data Availability

The data sets generated and analyzed during this study data are available from the Principal Investigator (JB) on reasonable request.
