# Supplementary material for: Promoting a Patient-Centered Understanding of Safety in Acute Mental Health Wards: A User-Centered Design Approach to Develop a Real-Time Digital Monitoring Tool
Source: JMIR Form Res. 2024 Apr 12;8:e53726. doi: 10.2196/53726 (PMC11053394; doi:10.2196/53726)
Supplement: Multimedia Appendix 1 [file formative_v8i1e53726_app1.pdf]

Multimedia Appendix 1. Evidence scan included articles.

	Citation
1	Barrera A, Gee C, Wood A, Gibson O, Bayley D, Geddes J. Introducing artificial intelligence in acute psychiatric inpatient care: qualitative study of its use to conduct nursing observations. BMJ Ment Health. 2020 Feb 1;23(1):34-8.
2	Melvin GA, Gresham D, Beaton S, Coles J, Tonge BJ, Gordon MS, Stanley B. Evaluating the feasibility and effectiveness of an Australian safety planning smartphone application: a pilot study within a tertiary mental health service. Suicide and Life-Threatening Behavior. 2019 Jun;49(3):846-58.
3	Dewa LH, Lavelle M, Pickles K, Kalorkoti C, Jaques J, Pappa S, Aylin P. Young adults' perceptions of using wearables, social media and other technologies to detect worsening mental health: A qualitative study. PLoS One. 2019 Sep 18;14(9):e0222655.
4	Christie S. Big White Wall: transforming mental health services through digital technologies. Mental Health and Social Inclusion. 2013 Nov 25;17(4):202-5.
5	Sheehan R, Hassiotis A. Digital mental health and intellectual disabilities: state of the evidence and future directions. BMJ Ment Health. 2017 Nov 1;20(4):107-11.
6	Marshall JM, Dunstan DA, Bartik W. Smartphone psychology: New approaches towards safe and efficacious mobile mental health apps. Professional Psychology: Research and Practice. 2020 Jun;51(3):214.
7	Hoffman L, Benedetto E, Huang H, Grossman E, Kaluma D, Mann Z, Torous J. Augmenting mental health in primary care: a 1-year study of deploying smartphone apps in a multi-site primary care/behavioral health integration program. Frontiers in psychiatry. 2019:94.
8	Bruen AJ, Wall A, Haines-Delmont A, Perkins E. Exploring suicidal ideation using an innovative mobile app-strength within me: the usability and acceptability of setting up a trial involving mobile technology and mental health service users. JMIR Mental Health. 2020 Sep 28;7(9):e18407.
9	Marshall JM, Dunstan DA, Bartik W. Smartphone psychology: New approaches towards safe and efficacious mobile mental health apps. Professional Psychology: Research and Practice. 2020 Jun;51(3):214.
10	Brimblecombe N, Quist H, Nolan F. A mixed-methods survey to explore views of staff and patients from mental health wards prior to introduction of a digital early warning system for physical deterioration. Journal of psychiatric and mental health nursing. 2019 Apr;26(3-4):65-76.
11	Bucci S, Berry N, Morris R, Berry K, Haddock G, Lewis S, Edge D. "They are not hard-to-reach clients. We have just got hard-to-reach services." Staff views of digital health tools in specialist mental health services. Frontiers in Psychiatry. 2019 May 10;10:344.
12	Tielman ML, Neerincx MA, Pagliari C, Rizzo A, Brinkman WP. Considering patient safety in autonomous e-mental health systems–detecting risk situations and referring patients back to human care. BMC medical informatics and decision making. 2019 Dec;19:1-6.
13	Depp CA, Mausbach B, Granholm E, Cardenas V, Ben-Zeev D, Patterson TL, Lebowitz BD, Jeste DV. Mobile interventions for severe mental illness: design and preliminary data from three approaches. The Journal of nervous and mental disease. 2010 Oct;198(10):715.
