# Supplementary material for: Promoting a Patient-Centered Understanding of Safety in Acute Mental Health Wards: A User-Centered Design Approach to Develop a Real-Time Digital Monitoring Tool
Source: JMIR Form Res. 2024 Apr 12;8:e53726. doi: 10.2196/53726 (PMC11053394; doi:10.2196/53726)
Supplement: Multimedia Appendix 2 [file formative_v8i1e53726_app2.pdf]

Multimedia Appendix 2.

Table S1. Conceptualization of safety: Synthesis of information from the systematic review, evidence scan, patient and health professional interviews and stakeholder engagement.

Systematic review (SR) and evidence scan (ES)	Patient interviews	Health professional interviews	Stakeholder engagement
• Risk sharing versus risk management definitions of safety exist (SR).<br>• Staff and patients may conceptualize safety differently, eg, staff may focus on risk, patients on relationships (SR).<br>• Restraint traumatic from patient perspective (SR).<br>• Patients' past experience of trauma on ward impacts their notions of safety (SR).	• Empathy, compassion and respect from staff important to create a feeling of safety.<br>• Patients calm and settled can create a feeling of safety.<br>• Behavior of other patients can make people feel unsafe.<br>• Lack of things for patients to do can contribute to feeling unsafe.<br>• How staff work together (ie, teamwork) linked to feeling safe or unsafe.<br>• Insufficient or inattentive staff linked to feeling unsafe. For example, staff not being proactive or reactive.<br>• Unpleasant atmosphere can be unsettling (eg, tension from an incident not dealt with properly/appropriately).<br>• Staff burnout, being overworked linked to feeling unsafe.<br>• Chaotic ward feels unsafe, such as when staff move around constantly or constantly fidgeting patients.<br>• Environment can have a positive and/or negative impact: locked building, cleanliness, private space, access to outdoor and green space, enough space, enough windows and natural daylight, outlook, day room next to office for surveillance.	• May depend on professional role, career stage, experience of previous safety incidents.<br>• Safety viewed as the responsibility of all staff.<br>• Safety related to having the skill set required to deal with incidents (eg, de-escalation and communication techniques).<br>• Safety related to working with knowledgeable and engaged staff.	• Safety might be assumed to be physical. Physical, emotional and mental aspects of safety are all relevant.<br>• COVID-19 context and potential for misinterpretation (eg, safety relating to masks and hand washing etc.).<br>• Asking about feeling safe or unsafe could create anxiety or introduce fear in someone who was hoping to be in a "place of safety". May not have previously thought they might feel unsafe.
**Implications for design and development phase**<br>➢ *Allow for a multifaceted conceptualization of safety.*<br>➢ *Consider how the word "safety" may be misinterpreted - provide a clear description.*			

Table S2. Anonymity: Synthesis of information from the systematic review and evidence scan, patient and health professional interviews and stakeholder engagement.

Systematic review (SR) and evidence scan (ES)	Patient interviews	Health professional interviews	Stakeholder engagement
• Importance of patient anonymity frequently mentioned. Patients need to express their vulnerabilities, honestly, safely, anonymously (ES).<br>• Confidentiality of information may be a concern, particularly when information input via electronic devices (ES).	• Positive about providing honest feedback, if anonymous.<br>• Information could be collected by a neutral representative to limit concerns about repercussions, to facilitate honest responses.	• Safety information fed through to staff needs to be anonymized so it cannot be linked to a particular patient.	• People need to feel comfortable providing feedback.

Implications for design and development phase

➢ *Anonymity essential.*

➢ *Reinforce feedback is anonymous and that everyone's experience is important - all feedback valued.*

➢ *Give clear information about the use and purpose of collecting information via digital technology.*

Table S3. Milieu (ward atmosphere), contagion and incidents: Synthesis of information from the systematic review and evidence scan, patient and health professional interviews and stakeholder engagement.

Systematic review (SR) and evidence scan (ES)	Patient interviews	Health professional interviews	Stakeholder engagement
• Patients aware of changes in the milieu and may be able to help plan appropriate interventions (SR).<br>• Need to assess the milieu frequently (SR).<br>• Need to consider contagion when developing technology that attempts to detect risk situations (ES).<br>• Tools and technologies need to recognize differences between forensic/non-forensic contexts and all male/all female wards (SR).	• Physical environment and cleanliness can affect the atmosphere on the ward.<br>• Recognition that it "builds up" prior to an incident.<br>• Incidents can cause a "chain reaction" or "knock on" effect.<br>• Perceived incidents as often being related to drugs and alcohol, and not being able to go outside to smoke, or not being able to get back on the ward (eg, from garden).<br>• Patients get treated the same yet all patients are different. At the same time some staff do their job, others don't.<br>• Incidents often start as something small and then escalate, particularly following a perception of unfairness.<br>• Poor leadership and staff shortages associated with increased numbers of incidents depending on who is "on duty".<br>• An earlier incident can lead to patients having a bad experience later that day.<br>• Described how poor staff attitude(s) can "pass onto" patients and the ward atmosphere.	• Influence of milieu and contagion recognized.<br>• Hard to articulate. Metaphors for contagion: ripples, pressure, domino effect; Metaphors for milieu: environment, atmosphere, a feeling.<br>• Agreement that an incident can lead to further incidents which can have a lasting effect on the ward over hours and days, as well as potential to transfer from shift to shift.<br>• Perceived incidents as often being preceded by micro-cues eg, "a look", someone walking with their "head down", a flurry of activity/noise, how space is being used, deviations from the norm, language and voice changes (patients and/or staff).<br>• Differences between night and day shifts.<br>• One person can upset a whole ward in part by diverting resources from other patients.<br>• Gender: contagion said to spread differently on male, female, and mixed wards.<br>• Use of restraints can contribute to the ward atmosphere and incidents.	• The idea of safety should be broad and include the traumatization of bystanders, as this is often not currently captured.

	• Strong sense of being able to perceive an unsettling or uneasy ward atmosphere.<br>• Male wards may be experienced as very violent.<br>• Staff appear to miss things/don't see everything.		

Implications for design and development phase

➢ *The technology needs to be sensitive to subtle changes in a negative direction in order to anticipate the potential for an incident occurring.*

➢ *Explore whether the technology can be sensitive to context eg, highlight when a safety incident has recently occurred.*

➢ *Location: potential for the technology to be sensitive to location within the ward eg, increased anxiety or input activity by patients into the technology (prior to and after an incident).*

➢ *Night and day: the technology needs to recognize night and day as different contexts.*

➢ *The technology may need to account for/recognize ward profiles (eg, all male, all female, mixed wards).*

➢ *The technology may need to account for/recognize differences between individual patients.*

Table S4. Digital technology in the ward: Synthesis of information from the systematic review and evidence scan, patient and health professional interviews and stakeholder engagement.

**Systematic review (SR) and evidence scan (ES)**	**Patient interviews**	**Health professional interviews**	**Stakeholder engagement**
• Training for staff important to increase confidence in using digital tools (ES).<br>• Use of ward champions when implementing a new initiative or intervention (SR).	• Positive views on mechanisms such as via mobile phone/an app.<br>• Some patients might need support with technology (eg, elderly patients).<br>• Some patients may be too ill to provide feedback. Can become hyper vigilant/take on as a responsibility.<br>• Preference to provide feedback privately, not publicly.<br>• More than one mechanism to provide feedback suggested.<br>• Flexible approach to frequency of giving feedback needed eg, pen and paper or talking directly to staff.<br>• Patients should be able to opt in or out.	• Divided opinion about digital technology on the ward.<br>• Variation between trust/ward infrastructure for digital technology (eg, Wifi, computers).<br>• Some patients might need support with technology/device.	• Some patients might not be familiar/able to use the technology.<br>• Some patients may be suspicious of the technology.<br>• Location of technology: one device or a reporting "point" could create negativity around the area it is placed and prevent people using it.<br>• A digital system could be/feel intrusive, and could feel impersonal.
**Implications for design and development phase**<br>➢ *Ensure the purpose of the monitoring tool is clearly described to patients and staff.*<br>➢ *The technology needs to be inclusive across a wide range of patients and digital abilities.*<br>➢ *Staff may need support with using the technology and with data interpretation.*<br>➢ *Consider multiple mechanisms for data collection other than a single device/reporting "point".*<br>➢ *Consider other relevant systems in place for staff.*			

Table S5. Involving patients in understanding safety: Synthesis of information from the systematic review and evidence scan, patient and health professional interviews and stakeholder engagement.

Systematic review (SR) and evidence scan (ES)	Patients interviews	Health professional interviews	Stakeholder engagement
• Potential for patient safety to be improved in acute mental healthcare settings when patients are involved in interventions (SR).<br>• When developing a questionnaire clear wording essential (SR).<br>• Expression through words and images (ES).<br>• Accessibility and inclusivity ie, people who do not speak English as their first language and different levels of literacy (ES).<br>• Flexibility to adjust measurement time points according to patient preference so not intrusive (SR).	• Have a sense of what is happening on the ward and being "more vigilant".<br>• Described there is a lot that goes on staff are not aware of.<br>• Potential for feedback to be collected at set times, or to be accessible at all times.<br>• Data collection – at least daily, but preference might vary by patient.<br>• Smiley face or thumbs up/down potential response options, or a scoring system ie, 0-10.	• Viewed patients as more sensitive to safety concerns (this can be both positive and negative).<br>• Type of ward (eg, male, female, mixed ward) impact on safety incidents and micro cues.<br>• Importance of collecting both quantitative and qualitative information.<br>• Suggestions: smileys, traffic light system, mixture of word, picture and sound clips.<br>• Data collection could be daily and sent to an app (for patients/staff) or a dash board/computer (for staff).<br>• Potential for numerical scores to be assigned to feelings of safety by patients.<br>• Not all patients speak English or have English as their first language.<br>• Why patients feel unsafe is also important.	• Safety words/synonyms: reassurance, security, comfortable, concerns, recovery, calm, protect, safeguard, caring, responsive.<br>• Relevance of how safety is described by other organizations eg, CQC – caring/uncaring, responsive and effective.<br>• Not everyone is able to express their views and feelings in words. Suggestion: traffic light system with an additional free text feature.<br>• Capture quieter voices – those who don't usually speak up.
**Implications for design and development phase**<br>➢ *Technology needs to incorporate the input of quantitative and qualitative data to give patients choice about the level of detail they provide.*<br>➢ *Explore using language/imagery that is universal and include a free text option to expand.*<br>➢ *Consider levels of providing feedback eg, level 1 reporting – a color or smiley face; level 2 – more detailed information.*<br>➢ *Explore whether thresholds need to be built into the system to trigger alerts/action.*<br>➢ *Consider how the phrases used to describe safety converge with those used by other relevant organizations.*<br>➢ *Consider the location of data collection and timings.*<br>➢ *Consider the implications of type of ward.*			

Note. CQC = Care Quality Commission.

Table S6. Feeding data back: Synthesis of information from the systematic review and evidence scan, patient and health professional interviews and stakeholder engagement.

**Systematic review (SR) and evidence scan (ES)**	**Patient interviews**	**Health professional interviews**	**Stakeholder engagement**
• Safety interventions can be difficult to implement in the context of a mental health ward environment if issues are beyond the control of staff (SR).<br>• Staff need clarity around what action/response is required (SR).<br>• Connectivity important for consistent functionality (ES).<br>• Importance of collaborative partnerships between researchers, clinicians, app developers, and patients to ensure data flow (ES).<br>• Digital tools need to be easily accessible and complement clinical practice (ES).<br>• Importance of early involvement of stakeholders when developing digital/mobile interventions (ES).	• Supported providing feedback if the information is used in a worthwhile way.	• Recognized any technology would be experimental initially.<br>• Suggestions for feeding data back:<br>- Live, continually and directly to staff whilst on the ward.<br>- Daily meetings (eg, huddles and handovers), weekly meetings, monthly meetings.<br>- Digital display boards (staff and patients).<br>- Direct patients to a bespoke website.	• The importance of responsiveness as reporting on feelings of safety is a vulnerable thing to do – if safety information is gathered it must be responded to in a timely way.<br>• Recognized the importance of how the technology is embedded.
**Implications for design and development phase**<br>➢ *The technology and mechanism of feeding data back needs to work within existing trust and ward infrastructure.*<br>➢ *Explore frequency/timing of monitoring the data to ensure staff can be responsive.*<br>➢ *Consider approaches to displaying the data.*<br>➢ *Consider who can see the data and when.*<br>➢ *Staff may need support with data interpretation and action.*			

Table S7. Unintended consequences: Synthesis of information from the systematic review and evidence scan, patient and health professional interviews and stakeholder engagement.

Systematic review (SR) and evidence scan (ES)	Patient interviews	Health professional interviews	Stakeholder engagement
• Digital tools shown to have high predictive validity may still require modifications according to patient feedback (SR).	• The responsibility of providing feedback may cause anxiety.	• Potential for this approach to create a “constant stress state” with implications for psychological and physical harm.	• Phrasing has the potential to create anxiety about the ward environment.<br>• Potential for digital feedback to be prioritized over verbal information as it is documented.
**Implications for design and development phase**<br>➢ *Important to provide choice and freedom on when to provide feedback.*<br>➢ *May need to offer different levels of providing feedback.*<br>➢ *Consider how the technology sits alongside verbal information and feedback.*			
