# Supplementary material for: Promoting a Patient-Centered Understanding of Safety in Acute Mental Health Wards: A User-Centered Design Approach to Develop a Real-Time Digital Monitoring Tool
Source: JMIR Form Res. 2024 Apr 12;8:e53726. doi: 10.2196/53726 (PMC11053394; doi:10.2196/53726)
Supplement: Multimedia Appendix 3 [file formative_v8i1e53726_app3.pdf]

Multimedia Appendix 3. Feedback following the ward visits and prioritisation.

Themes	As a (role)	I want (to do)	So that (goal)	Technical considerations and ideas for action	MoSCoW<br>Must have=M<br>Should have=S<br>Could have=C<br>Won't have (this time)=W
Conceptualization of safety	Patient	My world to be safe	I can get better	Introduction/guidance content	C
	CTM	Capture staff feelings of safety	Staff feel safe	Out of scope	S
Anonymity	Patient	The option to be anonymous	I don't have to describe why I feeling like that	Data will be anonymous and ward based	S
	Patient	Messages that are secure	I know where the information is going	Data will be anonymous and ward based	M
	Patient	Private messages	My feelings aren't shared on a board	Out of scope	S
	Patient	Use my name	Staff know it's me	Data will be anonymous and ward based	S
	Patient	To ensure my friends are ok on this ward	I don't have to worry about them	Data will be anonymous and ward based<br><br>Consider whether the system needs to share information between wards – linked to contagion/ripple effect	C
	Patient	To ensure my friends are ok on other wards	I don't have to worry about them	Data will be anonymous and ward based	C
Milieu (ward atmosphere), contagion and incidents	Staff	To observe the ripple effect	I can understand it better	Metrics/charts to help observe the milieu included in the dashboard	-
	Staff	It to be aware of shift patterns - day vs night	I can look at differences	Confirm exact data outputs/visualization<br><br>Access to historical data	-

	Staff	It to be aware of shift pattern/implications on staff		Confirm exact data outputs/visualization<br><br>Access to historical data	-
	Staff	To understand the potential difference between male/female patients	Awareness is better	Impact the deployment plan	-
Digital technology in the ward	Patient	To use touch screen	It's the same as my phone	Feedback via tablet device with touchscreen	S
	Patient	It to be chained/secured to the wall	It's not thrown at me	Impact the deployment plan	C
	Patient	To use my own phone, I have unlimited data	I have control	Input limited to specific tablet devices per ward	M
	Patient	To use my own phone, I have unlimited data	I don’t have to use slow computers	Input limited to specific tablet devices per ward	S
	Patient	Staff to use pagers	They are aware of the situation/triggers	Out of scope	C
	Patient	To use the internet	I can do things/look things up	Devices need to be locked down	C
	Staff	It to be in a booth	It isn't stolen/damaged	Impact the deployment plan	S
	Staff	There to be enough Wi-Fi for it to work	It can do all it promises	Impact the deployment plan	M
	Staff	The devices to be safe - ward phones get stolen	It is achievable	Impact the deployment plan	M
	Staff	To use a mobile device/phone	It is natural	Selection of technology	-
	Staff	Ensure there is adequate Wi-Fi	It works	Selection of technology	M
	Staff	No camera	So patients aren't worried about being recorded	Selection of technology/devices locked down	S
	Staff	Reassurances that I am not responsible for breakages/it being stolen etc.	I don't have to worry about devices on the ward	Impact the deployment plan	S
	Staff	Devices to be safe/ protected eg, boxed in	Everyone is safe, compliant with policies	Impact the deployment plan	M

	Staff	The tablet to be robust	I don't have to worry about them	Selection of technology	M
	Staff	Technology that people already know how to use	It is easy to use	Selection of technology	-
	Staff	It to be safe eg, the wards computers and lounge TV are housed		Impact the deployment plan	M
	Staff	No other functions	It can't be messed with	Devices to be locked down	S
	CTM	To lessen the impact of a large screen with only negative outputs	Patients and staff feel safe	Evidence for/against ward interface	S
Involving patients in understanding safety	Patient	To comment on noise and chaos of the ward	Staff can do something about it	Free-text input option about what it contributing to how a patient is feeling about the ward	S
	Patient	To understand current perceptions of safety	Concerns about COVID-19 safety are captured	Out of scope	-
	Patient	To say how I feel about other patients	I can say why the ward is like this	Out of scope	M
	Patient	A graded approach to inputs/outputs	I can express myself better	Need to confirm exact data outputs/visualization/impact the deployment plan	M
	Patient	It to express my mood now	Staff know how I'm feeling	Real-time	S
	Patient	To report on other things like there are no cups	I don’t become agitated about it	Free-text input option about what it contributing to how a patient is feeling about the ward	C
	Patient	To alert a member of staff to my safety that's more specific than the nurse call button	I can express my safety	Out of scope	C
	Patient	To be able to add things anytime of the day/night	It shows how I'm feeling	Real-time/impact the deployment plan	S
	Patient	It to capture individual concerns but also general ones	To give options	Free-text input option about what it contributing to how a	-

				patient is feeling about the ward	
	Patient	It to be easy/familiar to use	I don't have to learn new things	Simple English/easy to use	S
	Patient	To use my own words	I don't have to use "hospital" words	Simple English/easy to use	S
	Patient	To use patient based words	They describe feeling better eg, "worried", "scared", "fearful"	Simple English/easy to use	S
	Patient	Words that I use/no jargon	It has meaning for me	Simple English/easy to use	M
	Patient	Language that isn’t condescending	I feel like a person	Simple English/easy to use	M
	Patient	To use bright colors	They match the ward	Use illustrations/emoji’s/take into design consideration	C
	Patient	To input using pictures and words	I can express myself even when I'm distressed/have jumbled thoughts	Use illustrations/emoji’s	M
	Patient	One to one conversations with staff	I am heard	Impact the deployment plan<br><br>Introduction/guidance content	M
	Patient	Have some control over my time/responses	I don't feel pressured	Impact the deployment plan<br><br>Timeout of session - not too brief	S
	Patient	To put my feeling in when I want to	I can express myself when I want to	Impact the deployment plan	S
	Patient	It to use pictures as well as words	People who don't speak English can use it	Use illustrations/emoji’s	M
	Patient	Like to use my own words	I can say things how I want	Free-text input option about what it contributing to how a patient is feeling about the ward	C

	Patient	Use a drop down box of phrases	I don't always have to put my feelings into words	Free-text input option about what it contributing to how a patient is feeling about the ward	C
	Patient	It to maybe have an artificial intelligence element	I use a concept familiar to me	Out of scope	C
	Patient	To press a "suicidal" button	Staff are aware	Out of scope	C
	Patient	It to be explained properly - it is an optional extra NOT a replacement for human contact	It doesn’t replace human contact	Introduction/guidance content	-
	Patient	It to show the time as there are no clocks here	I know what time of day it is	Potential to include the time on the recording screen	M
	Staff	To also record positive responses also	It isn’t all negative	Feed into response options	M
	Staff	It to be used everywhere on the ward	So that people in isolation can access	Selection of device and how it is deployed. Fixed or mobile	M
	Staff	It to understand patient individual "norms"	Interventions are proactive/appropriate	Out of scope	-
	Staff	To record individual’s "usual" reactions to incidents	Plans/interventions can be improved	Out of scope	-
	Staff	Any pictures to be more than RAG (red, amber, green) eg, weather symbols or a rainbow color system	People aren't just ok, middle or not safe	Feed into illustration options	S
	Staff	Patients to be able to input when they like	We get a true sense of feelings	Impact the deployment plan	S
	Staff	Patients to be able to input then they want	They have a sense of mastery	Impact the deployment plan	S
	Staff	Patient to be able to record when they want to	They don’t have to "save" their views for groups or other processes	Impact the deployment plan	M
	Staff	Patients to have lots of opportunities to be heard	-	Impact the deployment plan	M

	Staff	To maybe use emoji’s	People are familiar and can express themselves	Use illustrations/emoji’s	C
	Staff	To be able to highlight poor environmental conditions eg, plaster coming off the wall	The environment is improved	Free-text input option about what it contributing to how a patient is feeling about the ward	C
	Staff	Patients to be able to say what they are feeling when they choose	They have choice	Impact the deployment plan	S
	Staff	The role of it to be fully explained to the patient	There are no detrimental effects on patients	Impact the deployment plan<br><br>Introduction/guidance content	-
	Staff	Understand how patients see/feel the ward as they "know exactly what’s going on"	I can learn the nature of the micro interactions	Evidence for/against ward interface	-
	Staff	Ensure data about all patients are captured	The "quiet" patient doesn't get ignored/parity of care	Decision for researchers	S
	Staff	Specifics about what is causing the unsafe feelings	I understand what is causing the feelings	Covered by the questions	-
	Staff	Patients who can’t read or write to be able to use it	Everyone can use it	Accessibility - simple English, icons, etc.	-
	CTM	To capture the feelings across the whole ward	Interventions are proactive	Impact the deployment plan	M
Feeding data back	Patient	It to flag to staff when there are issues	It doesn’t reduce time spent with staff	Staff dashboard /need to confirm exact data outputs/visualization	-
	Patient	To support used systems e.g., triangle of care	It enhances them	Need to confirm exact data outputs/visualization/impact the deployment plan	-
	Staff	To be sure I’m using the information	Patients don’t feel unheard - negative reinforcement	Operational	M

	Staff	To understand all the small complexities	I can recognize a pattern	Need to confirm exact data outputs/visualization<br><br>Access to historical data	-
	Staff	To use it to review the days/weeks data	We can reflect	Need to confirm exact data outputs/visualization<br><br>Access to historical data	-
	Staff	To use it to review with patients	It reflects feedback/collaboration	Need to confirm exact data outputs/visualization<br><br>Access to historical data	-
	Staff	Look at the information	I can use it to review with a patient or in groups	Need to confirm exact data outputs/visualization	M
	Staff	To be able to show Managers/CQC the information	Conditions are improved	Need to confirm exact data outputs/visualization	C
	Staff	To not rely on a scale to instigate a response	I don't lose my clinical reasoning skills	Need to confirm exact data outputs/visualization	C
	Staff	Outputs maybe in colors	It is graded to support care plans/discharge	Need to confirm exact data outputs/visualization	C
	Staff	To be able to see the whole ward and individuals in real time	I have a good overview of the environment	Real-time	S
	Staff	To know who else is aware	We work together	Need to confirm exact data outputs/visualization	S
	Staff	All staff to benefit/understand the system	Everyone is safe	Introduction/guidance content/need to confirm exact data outputs/visualization	-
	Staff	A tool that triggers reactions	Actions are proactive/we can plan	Need to confirm exact data outputs/visualization	-
	Staff	To use it in everyday established processes eg, safety huddle	We improve practice/embed it in culture	Impact the deployment plan	-

	Staff	To understand what I'm supposed to do with the information	My actions/care plans are right	Impact the deployment plan	M
	Staff	To use the system in established processes eg, PIPA, safety huddles, staff meetings	It has some meaning and we can explore reasons why and/or be proactive	Impact the deployment plan<br><br>Mutual help and huddle statements	M
	Staff	To use it to support safety plans	Interventions are collaborative	Impact the deployment plan	S
	Staff	To know how to respond	My actions are appropriate	Impact the deployment plan	C
	Staff	To see the same as patients	We are talking about the same things	Evidence for/against ward interface	S
	Staff	It to be viewable to all	We take a shared responsibility in the actions	Evidence for/against ward interface	M
	Staff	Everyone to be able to look at a large screen	Everyone is aware of what's happening	Evidence for/against ward interface	-
	Staff	Know what the data is collected for	I understand why we're doing it	Introduction on the staff dashboard - links to about the project	S
	Staff	Only see my wards information	I can concentrate on my ward	Each ward dataset needs to be separate	C
	Staff	To know if the displays are recorded	We can use them later for formulations	Access to historical data	S
	Staff	A longer term record of events	Debriefings/actions are effective	Access to historical data	-
	CTM	The outputs to be meaningful to staff	Handover/sharing of information is supported	Need to confirm exact data outputs/visualization	S
	CTM	Outcomes to be meaningful to support clinical interventions	Patients have better care/improved safety	Need to confirm exact data outputs/visualization	M
	CTM	Outcomes to be meaningful to support embedding into ward culture	-	Need to confirm exact data outputs/visualization	S

	CTM	To be able to see/read outputs	Ensure all staff are able to understand the outcomes/implications	Need to confirm exact data outputs/visualization	-
	CTM	Experienced staff to be able to demonstrate their clinical reasoning	Experience is shared and taught	Need to confirm exact data outputs/visualization	-
	CTM	To encourage less restrictive practice	Patients benefit	Impact the deployment plan	S
	CTM	It to support containment	It increases safety	Evidence for/against ward interface	C
	CTM	To encourage staff presence on the ward	Patients feel staff are available	Evidence for/against ward interface	M
	CTM	To support the ward culture of "containment"	Staff work cohesively	Evidence for/against ward interface	S
	CTM	Capture staff feelings of safety	Staff interventions are proactive	Out of scope	S
Implementation					
	Staff	Everyone to chip in	The tool is adopted	Easy to use	S
	Staff	Education on how to use it/technology	I can use it properly	Easy to use - simple so should not need "instructions"	-
	CTM	Staff engagement	Safety is better for staff and patients	Easy to use	S
Unintended consequences					

Note. CTM = Clinical Team Manager, CQC = Care Quality Commission, PIPA = Psychologically Informed Partnership Approach.
