# Supplementary material for: Promoting a Patient-Centered Understanding of Safety in Acute Mental Health Wards: A User-Centered Design Approach to Develop a Real-Time Digital Monitoring Tool
Source: JMIR Form Res. 2024 Apr 12;8:e53726. doi: 10.2196/53726 (PMC11053394; doi:10.2196/53726)
Supplement: Multimedia Appendix 4 [file formative_v8i1e53726_app4.pdf]

Multimedia Appendix 4. Technologies used and infrastructure.

**Patient recording interface**	Each ward has its own unique deployment of the core recording interface product with each ward having its own unique "instance". The recording interface is separate from other wards and accessible via a unique URL to ensure an easy-to-use experience for patents submitting data and to avoid increasing the number of questions asked eg, "which ward are you on?". Each ward can have has its own subdomain which is automatically generated using a random string of characters as the sub-domain. Eg, `{4-6 char}.wardsonar.co.uk`.<br>The instances of the product are stored in an S3 Bucket fronted with a Cloudfront distribution to handle caching and SSL termination. Route 53 DNS records have been updated automatically through a Cloudformation script which generates the unique sub-domain values. Secrets manager has also been used to store API keys, credentials and configuration values for the recording interface.
**Staff dashboard**	Each ward has their own staff dashboard independent of other wards, so no data can be exposed between wards. The dashboards are behind a layer of authentication and can only be accessed by authorized staff members with login credentials.<br>The staff dashboards are hosted directly within the Google Infrastructure, however, minor alterations to the digital partner's infrastructure were required in order to achieve a stable and secure connection from Google to their database:<br>• A read-replica MySQL database was deployed - this is a read-only and real-time version of the live database, but is exclusively used for data computation and reporting. Using a read-replica means these high resource intensive tasks do not affect the performance or stability of the master database.<br>• By default, the database within the infrastructure is not accessible publicly and is behind several layers of network security and firewalls. In order for the dashboards to connect, a secure link had to be created to allow Google servers to connect. This was achieved through whitelisting a set of IPs that belong to Google, which allowed access into Virtual Private Cloud (VPC) to connect to read-replica database instance.
**Public facing ward interface**	The ward interface communicates the output from a specific measure for public consumption.<br>The ward interface is a subsection of the Recording Interface product, accessed by appending/dashboard to the URL and thus uses the exact same technologies.<br>Using the same code base for the ward interface allowed us to entirely reuse code and develop the interface much quicker, especially as the existence of the Ward Interface product was unknown from the start and something that co-design informed.

**Application Programming Interface (API)**	The API is used to link all of the digital products together with the underlying data storage engine. It facilitates data recording, processing, representation and exporting. Where possible, we used an open data format standard so that the data can be interoperable with other systems/products (eg, JSON, REST, OpenAPI).<br>The API has a one public endpoint (/stats) which returns a single computed payload that is used to power the Ward Interfaces. The rest of the API endpoints require a simple authentication bearer token which is unique to each ward. This token identifies where submissions are coming from, as well providing an authentication layer.<br>• PHP 7.2 - The underlying programming language used to create the API<br>• Laravel 8.12 - An open-source application framework written in PHP that provides some reusable code for common tasks (eg, validation)<br>• MySQL 5.7 - The central database engine used where all data is stored and accessed<br>• PHPUnit: 9.3 - A library to help with automated testing of the API<br>The API is hosted on a dynamically sized group of application servers, fronted with a load balancer. All requests to the API first are forwarded to load balancer which then decides which application server to send the request to. This model allows for automatic scaling to handle traffic spikes.<br>The API code is packaged up into a Docker container (a unit of software which contains the application code and all of its dependencies) which is deployed to the application servers via the CI/CD pipeline Travis.<br>We make use of CloudFormation which allows most of the infrastructure to be templated and scripted, which automates all of the tasks of deployment.
