# Supplementary material for: Promoting a Patient-Centered Understanding of Safety in Acute Mental Health Wards: A User-Centered Design Approach to Develop a Real-Time Digital Monitoring Tool
Source: JMIR Form Res. 2024 Apr 12;8:e53726. doi: 10.2196/53726 (PMC11053394; doi:10.2196/53726)
Supplement: Multimedia Appendix 5 [file formative_v8i1e53726_app5.pdf]

Multimedia Appendix 5. WardSonar programme theory narrative description.

**Ward introductory meetings**	The implementation of the WardSonar monitoring tool includes start up meetings/visits to wards. These meetings address the rationale for development of a monitoring tool and discuss how the tool might help maintain and/or improve the safety of the ward by recognising patients as a key source of safety information in real time.<br>Staff will be trained in how to use the handheld devices to facilitate collecting feedback from patients, and on how to interpret the information available on the staff dashboard.<br>Relevant moderating factors:<br>• Engagement of, and support from senior management<br>• Engagement of, and support from ward staff<br>• Quality improvement capacity (individual staff and ward team)<br>• Quality improvement knowledge and capability (individual staff and ward team)<br>• Safety culture
**Patient feedback about safety measured in real time**	Staff invite patients to complete anonymous questions at multiple time points per day via the patient recording interface on the handheld device. This generates real time information about the patient perspective of safety/ward atmosphere. The introductory information provided to patients on the handheld device emphasises the purpose of the monitoring tool and that patients are recognised as a key source of safety information. This aims to generate a more complete understanding of safety as we know patients and staff may conceptualise safety differently (physical and emotional aspects of safety). Gender is also relevant here and the tool recognises differences between forensic/non-forensic contexts and all-male/all-female wards, as we know contagion may occur differently on male, female, and mixed wards.<br>Relevant moderating factors and mediators (proximal outcomes):<br>• Engagement of patients to provide feedback<br>• Engagement of, and support from ward staff<br>• Engagement of, and support from senior management<br>• Quality improvement capacity (individual staff and ward team)<br>• Quality improvement knowledge and capability (individual staff and ward team)<br>• Patient factors (eg, Gender, ethnicity, disorder, digital literacy)<br>• Individual staff factors (eg, Qualification, gender)<br>• Additional ward level factors (eg, Skill mix, staffing levels)<br>• Patients know their feedback about feeling safe is recognised
**Patient feedback**	The staff dashboard displays the information collected from patients in real time. How the information is displayed emphasises the current ratings from patients, as well as how this has changed since the last round of feedback was collected ie, the direction and information about

**about safety considered by ward staff to identify immediate issues and safety risks**<br><br>**Staff respond to immediate issues and safety risks, and monitor and improve the safety of the clinical environment**	what is making patients feel this way, to help direct staff in deciding on when and what action/intervention to take. At any given time this would support staff to be able to better anticipate and respond to shifts in the safety dynamic as close to real time as possible, which in turn may allow more effective management of the safety and quality of care via existing mechanisms.<br>This facilitates 'a collective awareness by staff of the workings of the service, and their ability to be sensitive and responsive to subtle changes and disturbances' as described in the MMS framework [16] definition of the domain sensitivity to operations which the monitoring tool was developed to sit within.<br>A negative ward atmosphere or an incident occurring may have a "ripple effect", having a more real time understanding of the feel of the ward aims to reduce the opportunity for behavioural and emotional contagion which may have a lasting effect on the ward over hours and days, as well as potential to transfer from shift to shift.<br>Relevant moderating factors and mediators (proximal outcomes):<br><br>• Quality of forum/meeting where patient feedback considered<br>• Multidisciplinary approach to reflect on safety data<br>• Quality improvement knowledge and capability (individual and ward team)<br>• Quality improvement capacity (individual and ward team)<br>• Staff perceived credibility and usefulness of patient feedback about safety<br>• Safety culture<br>• Engagement of, and support from ward staff<br>• Engagement of, and support from senior management<br>• Patients know their feedback about feeling safe is recognised<br>• Increased sensitivity to operations for monitoring and managing safety<br>• Shared, multidisciplinary understanding of patient perspective of feeling safe<br>• Real time understanding of safety<br>• Reduced opportunity for behavioural and emotional contagion resulting from a "negative" ward atmosphere directly, and via incident occurrence
**Relationship between mediators (proximal outcomes) and postulated distal outcomes**	Improved patient perceptions of safety and care experience (eg, psychological safety)<br>Reduced incidents - type and severity. May reduce: overall number of incidents; particular types of incidents; severity of incidents.<br>Improved safety or quality performance<br>Improved safety culture<br>Patient centred-service<br>Collaborative, multi-disciplinary approach to service improvement
